# Unilateral pedal lymphangiography plus computed tomography angiography for location of persistent idiopathic chyle leakage not detectable by ordinary contrast computed tomography

**DOI:** 10.1186/s12894-018-0323-x

**Published:** 2018-02-06

**Authors:** Dingyi Liu, Boke Liu, Weimu Xia, Qi Tang, Haidong Wang, Jian Wang, Yanfeng Zhou, Jiashun Yu, Wenmin Li, Mingwei Wang, Wenlong Zhou, Sang Hu, Yuan Shao

**Affiliations:** 1 Department of Urology, Department of Radiology, Shanghai Punan Hospital, Shanghai, People’s Republic of China; 2 Department of Urology, Shanghai Jiao Tong University Medical School Affiliated Ruijin Hospital, 197 Ruijin Er Road, Shanghai, 200025 People’s Republic of China; 3 Department of Urology, Chinese People’s Liberation Army Hospital 184, Yingtan, People’s Republic of China; 4 Department of Urology, Shanghai Post and Telecommunication Hospital, 666 Changle Road, Shanghai, 200040 People’s Republic of China; 5 Department of Urology, Shanghai Jiao Tong University Medical School Affiliated Ruijin Hospital North, 999 Xiwang Road, Shanghai, 201801 People’s Republic of China

**Keywords:** Lymphangiography, Chyluria, Computerized tomographic angiography, Precise location

## Abstract

**Background:**

To identify the value of unilateral pedal lymphangiography (LPG) plus computed tomography angiography (CTA) in accurate depiction of persistent idiopathic chyluria undetectable by ordinary contrast CT.

**Methods:**

Eighteen patients 44–63 years of age with persistent idiopathic chyluria who failed conservative management were included. Ordinary CT had not revealed a chyle leak. Cystoscopy, unilateral LPG, and post-LPG CT angiography (CTA) were sequentially performed. Ligation and stripping of the perirenal lymphatics were subsequently performed guided by lymphangiography and CTA.

**Results:**

LPG and post-LPG CTA detected 17 unilateral and one bilateral chyle leaks in the 18 patients, with clear images of the communication of lymphatic vessels and the renal collecting or vascular system. The success rate was significantly better than cystoscopy (100% vs 50.0%, *P* = 0.005) or LPG alone (100% vs. 72.2%, *P* = 0.016). Chyluria resolved after surgery in all patients; no relapses were found.

**Conclusions:**

LPG plus post-LPG CTA accurately characterized perirenal lymphangiectasia that was not demonstrated by routine contrast-enhanced CT or not suitable for magnetic resonance imaging. Despite of its invasiveness, this method is a good diagnostic alternative to LPG in patients with persistent chyluria requiring surgery.

## Background

Chyluria is the passage of chyle in the urine caused by the rupture of retroperitoneal lymphatics with leakage into the pyelocaliceal system, giving urine a milky appearance. The etiologies include thoracic duct stenosis, tuberculosis, cancer, trauma, pregnancy, filariasis, or the cause may not be clear. The result is dilatation of distal lymphatics and the eventual rupture of lymphatic vessels into the urinary collecting system [1, 2]. Although rare, severe fluid and protein loss may cause hypovolemia and hypoproteinemia in some patients. Cystoscopy, lymphangiography (LPG), computed tomography (CT), magnetic resonance imaging (MRI) and lymphoscintigraphy are used to diagnose and locate the origin of chyluria [2–4]. Combining LPG with post-LPG CT imaging may increase the ability to locate chyle leaks [2, 5, 6]., MRI is contraindicated in patients with implanted metal devices. Some patients fail conservative management because their chyle leaks are not visualized by routine contrast CT, and require additional evaluation of the lymphatic system before treatment can be started. The aim of this study was to evaluate the value of unilateral LPG with post-LPG CT angiography (CTA) in chyluria patients who failed to conservative management with chyle leaks undetectable by ordinary contrast CT.

## Methods

### Patients

Eighteen patients diagnosed with persistent idiopathic chyluria between January 2013 and March 2017 were included. Ten were men and eight were women. Their median age was 51.5 (range 44–63) years, and the duration of chyluria ranged from 3 to 30 years. No patient had a history of tuberculosis or trauma. The main clinical manifestations were recurrent milky urine and asthenia. Seven patients experienced edema, one experienced severe anemia, 16 had intermittent recurrent chyluria, and two had persistent chyluria. All patients had test-confirmed chyluria; three had chylous hematuria. All were negative for filariasis antibody, urinalysis showed no urinary tract infections, other diseases cancer, and trauma were excluded. Conservative treatments such as bed rest, plenty of water with limited fat intake, and renal pelvic instillation via retrograde ureteral perfusion, had all failed in these patients. Renal pelvic sclerotherapy with povidone iodine or dextrose was performed one or two times in 11 patients without effect. The sites of chyle leaks could not be visualized with routine contrast CT. Cystoscopy including at least 5 min of observations of both ureteric orifices found unilateral urinary excretion of chyle in only nine patients (six left and three right). LPG CTA was subsequently performed.

### LPG

The lymphatics were stained by injecting 2 ml methylene blue into the web space between the first and second toes of one foot. A linear cut-down was performed on the dorsum of the foot below the ankle 30 min later to isolate a lymphatic vessel. After cannulation of the lymphatic vessel with a 30 gauge needle, iodized oil (Lipiodol; Laboratoire Guerbet, Roissy, France), a contrast agent for LPG, was injected at a rate of 0.1 ml/min, not exceeding a total volume of 14 ml. CTA was performed immediately to assess apparent chyle leaks and to document the filling phase of the LPG.

### CTA

CTA was performed with a 320 × 0.5 mm detector row CT unit (Aquilion ONE, Toshiba, Japan). A 40 ml volume of Iobitridol or Omnipaque nonionic contrast medium, 350 mg/ml was administered via an antecubital vein by bolus injection at rate 3–4 mL/s using a power injector. Renal artery imaging scans were performed 20–30 s after injection, and scans of the urinary collecting system were performed after 3–5 min. Volume rendering (VR), maximum intensity projection (MIP) and multiplanar reformation (MPR) of the CT scans allowed accurate visualization of abdominal or retroperitoneal lymphatic vessels, lymphatic leakage, the kidney, renal artery, and the collecting system.

### Surgical treatment

Renal lymphatic stripping and ligation were performed in all patients based on the anatomic location indicated by the LPG and post-LPG CTA. Surgical approach was decided according to the patient’s condition, and both laparoscopy (4 cases) and open surgery (14 cases) were performed. A urine chyle test verified the therapeutic effect.

### Statistical analysis

Qualitative variables were compared using the χ2 test. *P*-values < 0.05 considered statistically significant. The statistical analysis was performed with SPSS 20.0 (SPSS Inc., Chicago, IL, USA).

## Results

**Fig. 1:**
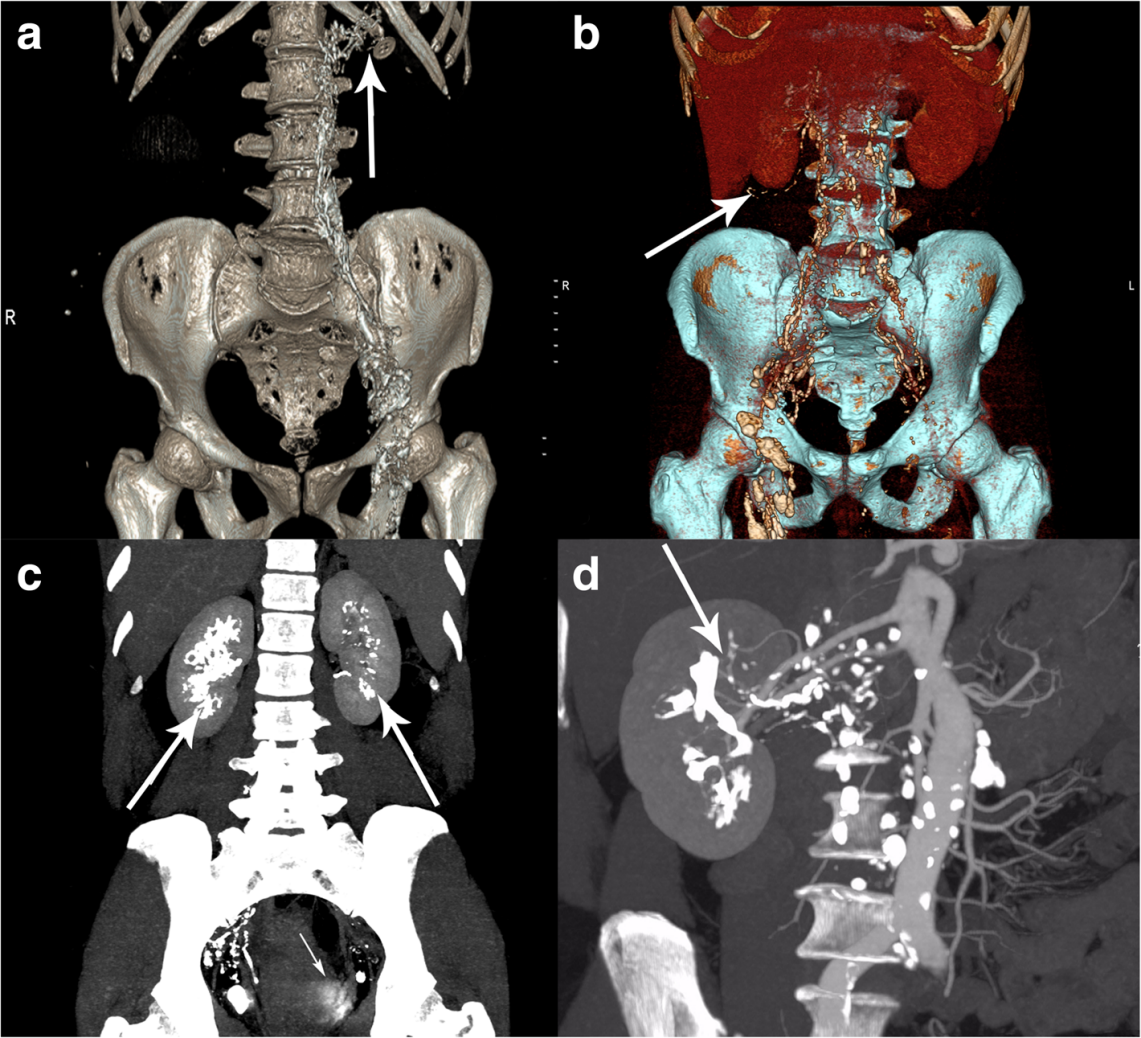
VR of CT data showing (**a**) the distribution of lymphatic vessels around the left renal artery and vein. **b** Chyle leaks in right renal fascia. MIP of CT data showing (**c**) Bilateral lymphatic leakage of the renal pelvis, and reflux of contrast agent into the bladder and (**d**) right renal lymphatic leakage and lymphatic vessel lesions adjacent to the renal vein

LPG and post-LPG CTA succeeded in delineating the thoracic ducts and confirming the absence of obvious obstructions. Ten of the 18 patients had lympho-urinary fistulas on the left side, seven were on the right side, nine of which were consistent with the cystoscopy results. The remaining patient was diagnosed with unilateral chyluria by cystoscopy, LPG found bilateral lesions. LPG plus CTA was more successful than cystoscopy in locating the side of chyle leaks (100% vs. 50.0%, *P* = 0.005, Table 1). When combined with post-LPG CTA, VR displayed the lymphatic reticular distribution in the kidney and renal fascia. In nine patients, VR and MIP revealed retroperitoneal lymphatic distortion, with reflux of lymphangiography contrast into the region of the contralateral iliac artery as well as showing the lymphatics adjacent to the renal arteries and veins (Fig. 1). LPG CTA provided detailed imaging information in all 18 patients. LPG alone provided imaging of equivalent value in only 13 patients. Accuracy of chyle leak location was better with LPG CTA than with LPG alone (100% vs. 72.2%, *P* = 0.016; Table 2). The results obtained with LPG CTA were sufficient to allow performing renal lymphatic stripping and ligation in all patients. Chyluria resolved immediately after surgery in 17 unilateral chyle leakage patients, with negative urine chyle tests. The remaining patient with bilateral lympho-urinary fistulas received renal lymphatic stripping on right side, which was the more severe side. His milky urine disappeared within 7 days after surgery, and a urine chyle test was negative. No recurrences were observed over a median follow-up 31 (range 8–52) months after surgery.

**Table 1:** The location of chyluria shown by cystoscopy and LPG CTA

Location of chyluria	Patient number	Cystoscopy	LPG + CTA	LPG	*p*-value*
Unilateral	17	9(52.9%)	17(100%)	17	0.0004
Left	10	6	10(58.8%)	10	
Right	7	3	7(41.2%)	7	
Bilateral	1	0	1	1	
Overall	18	9(50.0%)	18(100%)	18	0.005

*LPG* lymphangiography, *CTA* computerized tomographic angiography

*χ2 test

**Table 2:** Successful location of chyluria by cystoscopy, CT, LPG, and LPG CTA

	Cystoscopy	CT	LPG	LPG + CTA
Invasive	Y	N	Y	Y
Sides	Y/N	Y/N	Y	Y
Sites	N	Y/N	Y/N	Y
precise location	N	N	13/18	18/18*
Radiation	N	N	Y	Y**

*LPG* lymphangiography, *CTA* computerized tomographic angiography, *Y* Yes, *N* No

* *P* = 0.016 (LPG CTA vs. LPG)

** more radiation exposure than LPG or CT alone

## Discussion

Chyluria can be confirmed by a urine chyle test. About 80% of patients respond to conservative management with a low-fat, high-protein diet or intraperitoneal injection of sclerotherapy such as silver nitrate, povidone iodine or dextrose, about 22% relapse within 2 years [7, 8]. Surgical intervention is needed for patients with severe chyluria patients who fail to respond to conservative management or have short-term relapses. Successful surgery requires clear identification and accurate location of the site of chyle leakage, especially the relationship between lymphatic vessels and the renal collection system or vascular system.

**Fig. 2:**
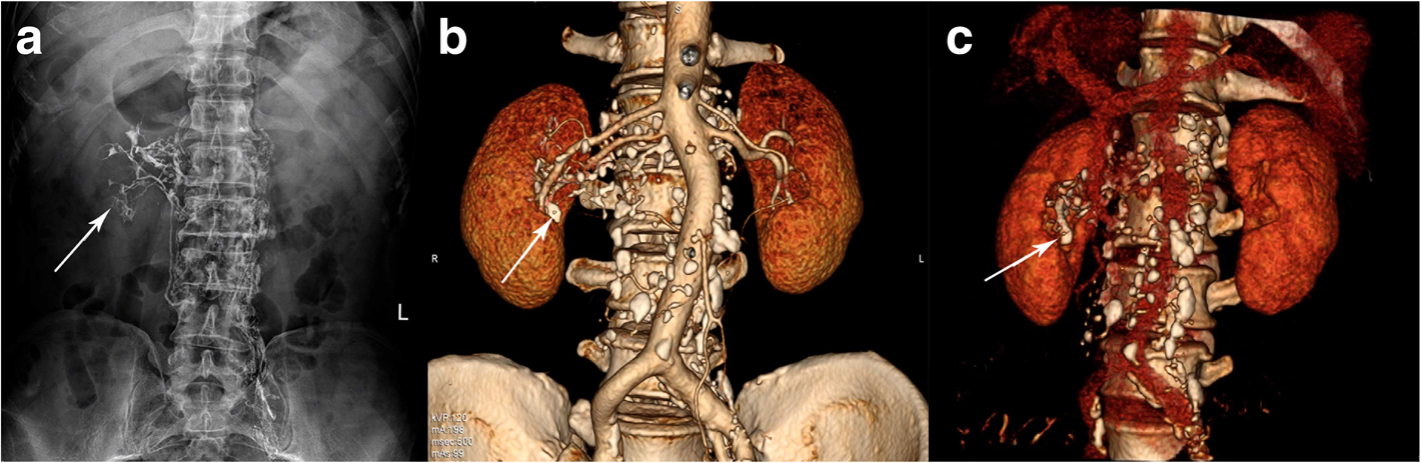
A 60-year-old man with persistent idiopathic chyluria. **a** Pre-CTA LPG showing wire-like, semicircular shadow at the renal pelvis area and parenchyma in LPG (KUB) indicated right renal lymphatic leakage. **b**, **c** LPG CTA showing accumulation of contrast agent in right renal hilus

Imaging studies in patients with severe chyluria generally include cystoscopy and LPG. LPG is more successful than cystoscopy in detecting bilateral lymphatic renal pelvis fistulas than cystoscopy [2]. The appearance of LPG images in these chyluria patients was wire-like, with semicircular or coralline-shaped shadows that were primarily distributed in the renal pelvis and parenchyma (Fig. 2a). The renal hilus was distorted and dilated, lumbar or iliac lymph vessels could be seen, and obviously dilated truncus lumbalis lymph vessels were occasionally observed. Single photon emission computed tomography (SPECT)/CT or MRI may be useful in the location of lymphatic ducts and chyle leakage sites [9–11], but LPG remains the most widely used method [12]. Evaluation of abdominal and retroperitoneal lymphatic abnormalities, including lymphatic leaks, using MRI lymphography with heavily T2-weighted fast spin echo sequences [10]. Nonenhanced MRI lymphangiography is a safe and effective method for imaging the central lymphatic system, and can contribute to differential diagnosis and appropriate preoperative evaluation of chylothorax or lymphangioma [13]. However there been few reports have described its use in chyluria patients [14].

**Fig. 3:**
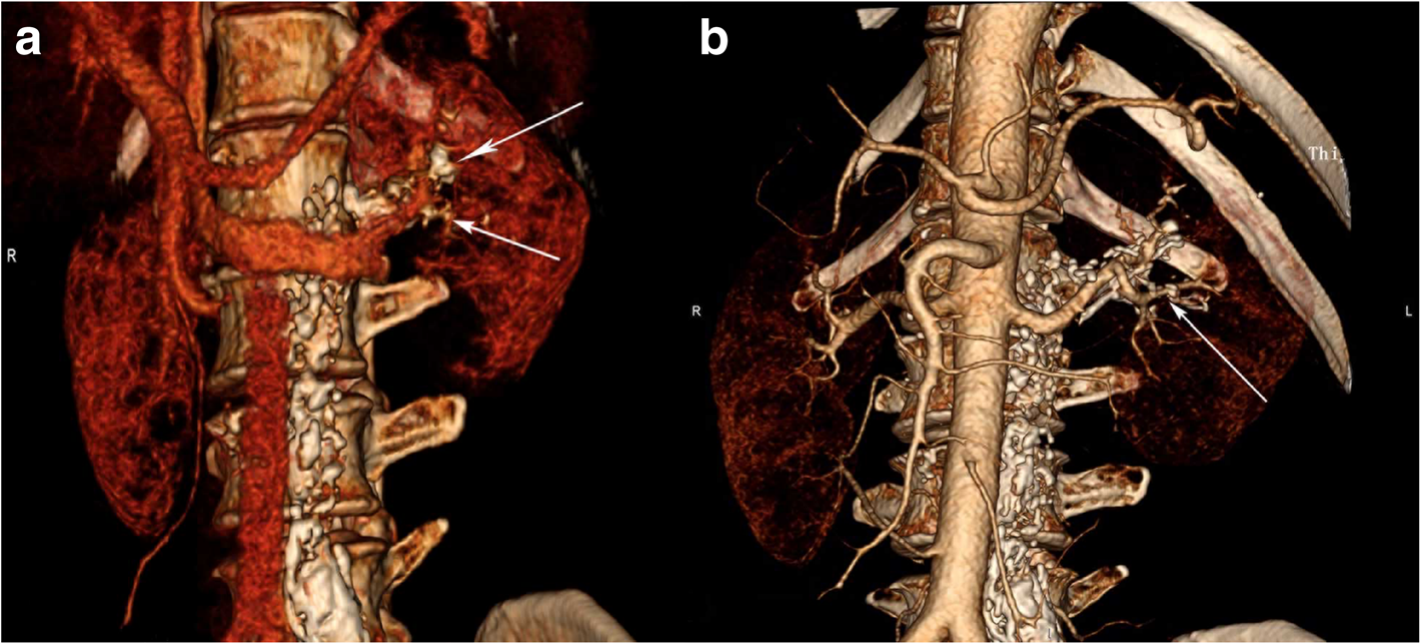
A 61-year-old woman with persistent idiopathic chyluria. **a**, **b** LPG CTA showing contrast agent adjacent to the left renal artery and its branches into the kidney

We previously reported the successful use of LPG in diagnosing chyluria and LPG followed by a CT scan to directly show fistulae between the perinephric collection and lymphatic systems in either a plain scan or reconstructed image [2]. It is not clear whether a CT scan is of help after LPG. The CT increases the radiation exposure, and may not provide the information needed to perform the required surgery. It cannot show the details of the connections between lymphatic vessels and renal blood vessels, which may result in surgical failure because of incomplete ligation of all the lymphatic branches surrounding the renal arteries or veins. LPG combined with post-LPG CTA clearly show such structures and the relation of the lymphatic vessels and the renal collecting or vascular systems. In this study, the lymphatic lesions were well visualized by LPG with post-LPG CTA in all patients (Figs. 2 and 3), providing a reliable basis for renal pedicle lymphatic ligation and stripping.

Cystoscopy correctly found the side of the chyle leak in only about half the patients. LPG, the classic diagnostic tool [12], revealed not only the side of the lymphatic leaks, but also the approximate sites of reflux of contrast agent reflux into the renal collecting system (see Fig. 2a). However, LPG was unsatisfactory in some complicated cases, and it was difficult to obtain more information on the chyle leak in addition to the side of chyluria. LPG combined with post-LPG CTA clearly showed additional detail including the course of renal blood vessels, lymphatic vessels, the collection system, and their interlaced connections. The radiation exposure was more with LPG CTA than with LPG alone (Table 2), but in complicated cases in which CT or LPG alone were not satisfactory, LPG combined with post-LPG CTA provided precise location and clear imaging information, especially in cases not suitable for use of MRI. Despite its level of invasiveness, this method is a good option in the diagnosis of persistent chyluria requiring surgery. Fever and pain are the most frequent complications after LPG. Severe complications such as hemoptysis, wound infection, and embolism of blood vessels have been reported [12, 15, 16], but did not occur in this patient series.

The study was limited by a small number of patients because of the rarity of persistent idiopathic chyluria and by the absence of a randomized control group. A multicenter, randomized control study with large number of patients is necessary to further investigate the advantages of LPG with CTA in the accurate location of chyle leaks and the management of chyluria.

## Conclusions

LPG combine with post-LPG CTA could provide precise location and clear imaging information for chyluria patients which cannot be detected by routine contrast CT or not suitable for MR examination. Despite of its mild invasiveness, this method could be better than LPG alone and be a good option in the diagnosis of persistent chyluria requiring surgery.

## Abbreviations

CT: Computed tomography
CTA: Computerized tomographic angiography
LPG: Unilateral pedal lymphangiography
MIP: Maximum intensity projection
MPR: Multiplanar reformation
MRI: Magnetic resonance imaging
SPECT/CT: Single photon emission computed tomography/computed tomography
VR: Volume rendering
